# *In vivo* exposure to bisphenol F induces oxidative testicular toxicity: role of Erβ and p53/Bcl-2 signaling pathway

**DOI:** 10.3389/frph.2023.1204728

**Published:** 2023-08-02

**Authors:** Adeyemi Fatai Odetayo, Wale Johnson Adeyemi, Luqman Aribidesi Olayaki

**Affiliations:** ^1^ Physiology Department, University of Ilorin, Ilorin, Nigeria; ^2^ Physiology Department, Federal University of Health Sciences, Ila Orangun, Nigeria; ^3^ Physiology Department, Adeleke University, Ede, Nigeria

**Keywords:** bisphenol f, endocrine disruptors, oxidative stress, hypothalamic-pituitary-gonadal axis, inflammation, apoptosis

## Abstract

**Introduction:**

Bisphenol F (BPF), an alternative to bisphenol A has been implicated as a gonadotoxic substance. BPF has been shown to induce hormonal imbalance and testicular oxidative damage. However, the mechanism associated with BPF-induced testicular toxicity has not been fully explored. This study was designed to explore the role of tumor protein (p53)/ B-cell lymphoma 2 (BCl-2) signaling and oestrogen receptor beta (Erβ) in BPF-induced testicular toxicity.

**Methods:**

Male Wistar rats were randomized into control (Cntrl), BPF-treated (10, 30, and 50 mg/kg for low dose (BPF-L), medium dose (BPF-M), and high dose (BPF-H) respectively), and BPF-treated recovery (Cntrl-R, BPF-L-R, BPF-M-R, and BPF-H-R). The administration was via gavage and lasted for 28 days and the animals in the recovery groups were allowed 28-days exposure free period for recovery from BPF exposure.

**Results:**

BPF resulted in the distortion of the testicular histoarchitecture, which was accompanied by a significant rise in testicular gamma-lutamyl transferase and lactate dehydrogenase activities but a decline in sorbitol dehydrogenase activities. Also, BPF caused a significant reduction in plasma gonadotropin-releasing hormone, luteinising hormone, follicle-stimulating hormone, and testosterone, which was associated with the downregulation of testicular 3beta-hydroxysteroid dehydrogenase and 17beta-hydroxysteroid dehydrogenase activities. Furthermore, BPF induced testicular inflammation, redox imbalance, and apoptosis, accompanied by distortion in p53/BCl-2 signaling and overexpression of Erβ. Again, the observed toxic effects of BPF were dose-dependent and not completely reversed by BPF cessation.

**Discussion:**

Bisphenol F induced gonadotoxicity by distorting p53/BCl2 signaling and the expression of Erβ. These observed alterations were not completely reversed after the cessation of BPF exposure.

## 1. Introduction

Male infertility is commonly caused by medications, disease conditions, genetic mutations, negative lifestyle modifications, and environmental pollutants (1). Recently, people have been more concerned about male infertility that is caused by environmental contaminants such as heavy metals, radiation, and organic chemicals such as bisphenols (2).

Bisphenol F (BPF) is one of the most commonly used alternative chemicals in place of bisphenol A (BPA). The chemical has been detected in individuals' serum, urine, and breast milk in industrialised countries (3). Plastics and cans, commonly used for packaging food and drinking water, are one of the major sources of BPF exposure (4). In addition to the dietary route, humans can also be exposed to BPF via dermal contact and inhalation. Human exposure to BPF has been associated with hormonal imbalance in males (5), poor sperm quality and oxidative stress (6), and apoptosis (7). However, the mechanism behind BPF-induced apoptosis is yet to be fully explored. The role of the p53/Bcl-2 signaling pathway in BPF-mediated testicular toxicity remains unclear. Furthermore, the contribution of Erβ in BPF-induced testicular toxicity has not been unraveled.

Tumor protein p53 (p53) is often referred to as the “guardian of the genome” due to its ability to protect the cell from biological and environmental insults that can lead to cancer. In fact, early studies have implicated mutations that disrupt the functions of p53 as drivers of cancer (8). On the other hand, B-cell lymphoma 2 (Bcl-2) is an antiapoptotic protein (9) with a specific relationship with p53. An over-expression of p53 has been shown to cause apoptosis either by directly inhibiting the activities of Bcl-2 (10) or inducing Bax transcription, which in turn overwhelms the antiapoptotic effect of Bcl2 (11).

Besides the influence of p53 and Bcl-2 on the apoptotic process, oestrogen receptors also play a dominant role. Similar to oestrogens, oestrogenic compounds such as BPF act by binding with oestrogen receptors. Oestrogen receptors found in the nucleus of cells are of two subtypes, viz; oestrogen receptor α and β (ERα and ERβ). While ERα promotes cell proliferation, Erβ prevents cell growth and stimulates apoptosis. Specifically, oestrogen maintains spermiogenesis via ERα while it regulates spermatocyte apoptosis via Erβ. Erβ suppresses tumour growth via the pro-apoptotic function (12). The oestrogen receptor subtype has also been shown to contribute to the maintenance of apoptotic markers such as IAP, BAX, caspase-3, and PARP and to stimulate the NF-κB/BCL-2 signaling pathways, which mediates apoptosis in tumours (12). Apart from the role of Erβ on apoptosis, the receptor has also been shown to stimulate autophagy by inhibiting the PI3K/AKT/mTOR signaling and stimulating the AMPK pathway (12).

Although there are reports on the pro-oxidative effect of BPF; however, there is no report on the modulation of the p53/Bcl-2 signaling pathway and Erβ as part of the mechanisms by which BPF-induced testicular toxicity. Hence, the present study investigated the role of this pathway in BPF-enhanced gonadotoxicity and the implications of Erβ and NF-κB. Moreover, we investigated the possibility of reversed effects of BPF after cessation of exposure.

## 2. Experimental design

### 2.1. Chemical/reagents

Assays were done using standard ELISA and colourimetry kits according to the manufacturers' guidelines as well as established colourimetry methods. The constituents of the phosphate buffer solution (PBS) used in this study include 98% pure sodium phosphate anhydrous dibasic salt (LOBA Chemie PVT. LTD, CAS NO: 7558-79-4 ADR/PG), 99% pure sodium dihydrogen orthophosphate monohydrate (LOBA Chemie PVT. LTD, CAS NO: 10049-21-5 ADR/PG) and 37% hydrochloric acid fuming (EMSURE® ACS, ISO, Reag. Ph Eur, 1.00317.2500). All other reagents used, including BPF (CAS: 620-92-8), except otherwise stated, were of analytical grade and procured from Sigma-Aldrich, USA.

### 2.2. Animals

Forty-eight (48) male Wistar rats of comparable age and weight (10 ± 2 weeks, 180 ± 20 g) purchased from the Biochemistry Department, University of Ilorin, were housed in standard well-ventilated wood cages (*n* = 6 rats/cage). The rats were given unlimited free access to food and water and a 12-h cycle of light and darkness. The animals were handled carefully based on the Guidelines for Laboratory Animal Care published by the National Institute of Health (NIH). The experimental protocol was under the US NAS guidelines, and ethical approval was obtained from the University of Ilorin Ethical Review Committee (UERC).

### 2.3. Experimental procedure

Animals were randomly divided into eight groups of six rats each, as indicated below:

Group 1: Control (Cntrl), vehicle-treated with 0.5 ml of corn oil
Group 2: Low-dose BPF (BPF-L), received 10 mg/kg b.w of BPF
Group 3: Medium-dose BPF (BPF-M), received 30 mg/kg b.w of BPF
Group 4: High-dose BPF (BPF-H), received 50 mg/kg b.w of BPF
Group 5: Control recovery (Cntrl-R), received 0.5 ml of corn oil followed by 28-days of no administration of corn oil.
Group 6: Low-dose of BPF recovery (BPF-L-R), received 10 mg/kg b.w of BPF followed by 28-days of no administration of BPF
Group 7: Medium-dose BPF recovery (BPF-M-R), received 30 mg/kg b.w of BPF followed by 28-days of no administration of BPF.
Group 8: High-dose BPF recovery (BPF-H-R), received 50 mg/kg b.w of BPF followed by 28-days of no administration of BPF.

The dose of 10 mg/kg used in the present study was chosen as a lower dose than the no-observed-effect level of 20 mg/kg reported earlier (13), while the doses of 30 mg/kg and 50 mg/kg were chosen as higher doses to the no-observed-effect level reported (13). The doses used in the current study are similar to the doses used and reported by Ullah et al. (14). The dose of BPF was calculated and dissolved in corn oil, and 0.5 ml of the solution, containing the appropriate calculated dose, was administered for each animal. All administrations were once daily via the oral route using an oro-pharyngeal cannula for 28 days (4). The doses used in this research are similar to previous studies (13 14).

The animals were sacrificed 24 h after the administration of BPF on the last day of the experiment. However, animals in the recovery groups were sacrificed 28 days after the termination of the non-recovery aspect of the experiment (15). The length of the recovery period is the same as the treatment period (4). Animals were sacrificed via intraperitoneal administration of ketamine (40 mg/kg) and xylazine (4 mg/kg) (16, 17). Blood samples were collected through the cardiac puncture, centrifuged at 3,000 rpm for 10 min using eppendorf centrifuge 5,415 D, and the plasma was separated for biochemical assays. Both testes were harvested, and surrounding tissues were removed. The left testes were homogenized in phosphate buffer for biochemical assays, while the right testes were preserved with 10% formaldehyde for histology and immunohistochemistry.

### 2.4. Histological preparation and scoring

Testicular histopathological analysis was carried out as previously reported (16). The testis was fixed in bouine solution, dehydrated with ethanol series, and cleared with toluene. The testes were embedded at room temperature and blocked in paraffin wax incubated overnight in a 60°C incubator. Afterward, hematoxylin and eosin (H&E) stain was applied to the testes' 5 µm thick paraffin sections.

For histological scoring, twenty seminiferous tubules were counted per slide and the data obtained were normalised to control. The first field was selected at random, while the subsequent fields were chosen by clockwise movements. Rounded seminiferous tubules at the similar phase of spermatogenic cycle were chosen. Also, the histopathological analysis and testicular planimetry were conducted by two experts who were blinded to the study protocol and the average value for each parameters were used. The testicular planimetry was determined using ×100 magnification, while ×400 was used for cell count.

Testicular histoarchitecture and mean testicular biopsy score were determined as earlier established by (18, 19) scoring systems respectively using Image J Software (US National Institutes of Health, Bethesda, MD, USA). Also, the Leydig cell mass, mean seminiferous tubular and luminal diameter, epithelial height, and Sertoli cell number were determined as earlier established (18, 20) using Image J Software (US National Institutes of Health, Bethesda, MD, USA). The cell count was done manually, and the Sertoli cells were identified as cells with pale nuclei within the seminiferous tubules close to the basement or tunica albuginea. In contrast, the Leydig cells were identified as cells within the interstitial space. Although GATA4 staining is the standard for identifying Sertoli cells, H&E could be used.

### 2.5. Measurement of circulatory hormones

The plasma concentration of gonadotropin releasing hormone (GnRH) (Melsin, China; catalog no: EKRAT-0426), follicle-stimulating hormone (FSH) (Bio-Inteco, UK; catalog no: B-1-121040801), Luteinising hormone (LH) (Bio-Inteco, UK;catalog no: B-1-121032301), and testosterone (Bio-Inteco, UK; catalog no: B-1-121071602) were estimated following the instruction from the manufacturers using a spectrophotometer.

### 2.6. Determination of steroidogenic enzymes

The effect of BPF on testicular 3 beta-hydroxysteroid (3β-HSD) and 17 beta-hydroxysteroid (17 β-HSD) were estimated according to the method of (21, 22), respectively.

For 3β-HSD, testicular tissue was homogenized, and the supernatant was carefully separated. 1 ml of the supernatant was mixed with 1 ml of 100 μmol sodium pyrophosphate buffer (pH 8.9), 30 μg of dehydroepiandrosterone in 40 μl of ethanol, and 960 μl of 25% BSA. The mixture was then incubated and 0.5 μmol of NAD was added. The absorbance was read spectrophotometrically at a wavelength of 340 nm using a blank as reference. For testicular 17β-HSD, 1 ml of the supernatant obtained from the testicular sample was mixed with 1 ml of 440 μmol sodium pyrophosphate buffer (pH 10.2), 40 μl of ethanol containing 0.3 μmol of testosterone, and 960 μl of 25% BSA. The mixture was incubated and 1.1 μmol of NAD was added in a U 2,000 spectrophotometer cuvette at 340 nm against a blank.

According to the manufacturer's instruction (Randox, UK, LOT: 239991, plasma cholesterol, the precursor of testosterone, was estimated using a standard ELISA kit with a spectrophotometer.

### 2.7. Estimation of testicular enzymes

Gamma-glutamyl transferase (GGT) activities were estimated according to the manufacturer's instructions (Agape Diagnostics Ltd., CAT: 31070095), while Lactate dehydrogenase activities were also determined following the manufacturer's instructions (Agape Diagnostics Ltd., CAT: 31060230) using a spectrophotometer. The testicular lactate concentration was also estimated according to the manufacturer's guidelines (EnzyChrom, ELAC-100).

### 2.8. Measurements of markers of inflammation

Standard ELISA kits were used to assay the concentrations of interleukin-6 (IL-6) (Solarbio, China, CAT: SEKH-0013) and tumour necrosis factor-α (TNF- α) (Solarbio, China, CAT: SEKH-0047), NFkB (Elabscience Biotechnology Inc., USA, CAT: E-EL-R0673) in the testicular tissue following the manufacturers' guidelines using a spectrophotometer. Testicular myeloperoxidase (MPO) was determined based on established principles (18, 23). This method involves guaiacol oxidation in the presence of hydrogen peroxide. As previously documented, testicular nitric oxide was evaluated based on the Griess reaction (24).

The method for measuring MPO is based on the ability of myeloperoxidase to catalyze the oxidation of guaiacol to oxidized guaiacol in the presence of hydrogen peroxide. The oxidized form of guaiacol has a brown color, which is measured spectrophotometrically at a wavelength of 470 nm. The intensity of the color produced is proportional to the concentration of oxidized guaiacol produced in the reaction, thus providing a measure of myeloperoxidase activity.

For NO, a mixture of 100 μl of Griess reagent, 300 μl of a nitrate-containing testicular homogenate, and 2.6 ml of deionized water were incubated for 30 min at room temperature in a spectrophotometer cuvette. A blank was prepared by mixing 100 μl of Griess reagent and 2.9 ml of deionized water. The absorbance of the nitrate-containing sample was measured at 548 nm in relation to the reference sample.

### 2.9. Antioxidant enzymes and testicular oxidative stress measurement

Malondialdehyde (MDA), a marker of oxidative stress, was determined as previously documented (18, 25) based on the generated amount of thiobarbituric acid reactive substance (TBARS) during lipid peroxidation.

According to Ellman's method, the testicular glutathione (GSH) level was estimated. Also, catalase (CAT), glutathione peroxidase (GPx), Glutathione-S-transferase (GST), and testicular superoxide dismutase (SOD) activities were evaluated by the colorimetric method through the usage of standard laboratory reagents (26).

Malondialdehyde (MDA), a marker of oxidative stress, was determined as previously documented based on the generated amount of thiobarbituric acid reactive substance (TBARS) during lipid peroxidation. This method involves the reaction between 2-thiobarbituric acid (TBA) and malondialdehyde, a byproduct of lipid peroxidation, by analyzing the pink chromogen complex [(TBA) 2-malondialdehyde adduct] formed upon heating at acidic pH. The sample (200 μl) was first treated with 500 μl of Trichloroacetic acid (TCA) to remove proteins and centrifuged at 3,000 rpm for 10 min. Next, 1 ml of 0.75% TBA was added to 0.1 ml of the supernatant and heated in a water bath at 100°C for 20 min, then cooled with ice water. The absorbance of the sample/standard was then read at 532 nm using a spectrophotometer and compared to a blank. The concentration of TBARS was determined by extrapolating from a standard curve.

For GSH, an aliquot of the sample was deproteinized by adding an equal volume of 4% sulfosalicylic acid, and was centrifuged at 4,000 rpm for 5 min. 0.5 ml of the supernatant was then added to 4.5 ml of Ellman's reagent. A blank was prepared by mixing 0.5 ml of the diluted precipitating agent with 4.5 ml of Ellman's reagent. The level of GSH was calculated by measuring the absorbance at 412 nm.

For catalase, 1:29 dilution of the sample was made by mixing 1 ml of the supernatant of the testicular homogenate with 19 ml of diluted water. 4 ml of H2O2 solution (800 μmoles) and 5 ml of phosphate buffer were added to a 10 ml flat bottom flask. 1 ml of the diluted enzyme preparation was mixed into the reaction mixture by gentle swirling at 37°C. Samples of the reaction mixture were withdrawn at 60 s intervals, and the H2O2 content was determined by blowing 1 ml of the sample into 2 ml dichromate/acetic acid reagent. Catalase levels in the sample were determined by comparing the absorbance at 653 nm to that of a certified catalase standard.

For GPx, the sample was incubated at 37°C for 3 min, then 0.5 ml of 10% trichloroacetic acid (TCA) was added and the mixture was centrifuged at 3,000 rpm for 5 min. The supernatant was then mixed with 2 ml of phosphate buffer and 1 ml of 5′-5′- dithiobis-(2-dinitrobenzoic acid (DTNB) solution, and the absorbance was measured at 412 nm using a blank as reference. The GPx activity was determined by plotting a standard curve and determining the concentration of remaining GSH from the curve.

The activity of glutathione-S-transferase in testicles was also measured. This method utilizes the enzyme's high activity with 1-chloro-2,4-dinitrobenzene as a substrate. The assay was performed at 37°C for 60 s and the absorbance was read at 340 nm after comparing it with a blank sample.

For SOD, a 1:10 dilution of the sample was made using 1 ml of sample and 9 ml of distilled water. 0.2 ml of the diluted sample was added to 2.5 ml of 0.05 M carbonate buffer (pH 10.2) and the reaction was initiated by adding 0.3 ml of freshly prepared 0.3 mM adrenaline. The mixture was mixed and the increase in absorbance was monitored at 480 nm every 30 s for 150 s using a spectrophotometer. A reference cuvette containing 2.5 ml buffer, 0.3 ml substrate (adrenaline), and 0.2 ml water was also used.

### 2.10. Xanthine oxidase and uric acid measurement

Testicular xanthine oxidase (XO) activities were estimated using the method of (27), while the concentration of testicular uric acid (UA) was estimated using colorimetric methods based on standard laboratory kit (Precision, UK) using a spectrophotometer.

### 2.11. Estimation of apoptotic markers

As previously described, a spectrophotometric assay using diphenylamine (DPA) methods (18, 28) was employed to determine the estimated percentage of DFI. 5 ml each of testicular homogenate supernatant and pellet were treated with 3 ml of freshly prepared diphenylamine (DPA) reagent for colour development. The solution was incubated at 37°C for 16 to 24 h. The absorbance of light green/yellowish-green supernatant was read spectrophotometrically at 620 nm. The percentage of fragmented DNA was calculated by dividing the absorbance of the homogenate supernatant by the sum of the absorbance of the pellet and the absorbance of the supernatant.

Testicular Caspase 3 activities were estimated according to the manufacturer's instructions (Elabscience Biotechnology Co., Ltd., USA), while testicular 8-OHdG was determined using the competitive-ELISA principle according to the manufacturer's guidelines (Elabscience Biotechnology Co Ltd, USA with catalog no: E-EL-0028).

### 2.12. Immunohistochemistry analyses for Erβ, p53 and Bcl-2 expression

Formalin-fixed and paraffin-embedded testicular tissues were sectioned at 4 μm for immunohistochemistry. Immunohistochemical procedures were performed using appropriate antibodies; anti-mouse Erβ monoclonal for Erβ expression (1:100), anti-mouse p53 monoclonal for p53 expression (1:100), and anti-mouse Bcl-2 monoclonal for Bcl-2 expression (1:200). The formalin-fixed and paraffin-embedded testicular tissues were sectioned at 4 μm for immunohistochemistry. Appropriate antibodies; anti-mouse Erβ monoclonal for Erβ expression (1:100) (Leica Biosystems, USA with CAT NO: 6069100), anti-mouse p53 monoclonal for p53 expression (1:100) (Espredia with CAT NO: 186P2105D), and anti-mouse Bcl-2 monoclonal for Bcl-2 expression (1:200) (Thermo Fisher Scientific, USA) (29). Shortly, after de-paraffinization and rehydration of sections, the antigen was retrieved using preheated citrate buffer and allowed to cool for 30 min. The slides were cleaned with Kim wipes, section areas marked with a hydrophobic pen, and slides were then arranged in a humidified chamber. The slides were incubated for 10 min following blockade of endogenous peroxidase activity using hydrogen peroxide. The slides were rinsed with phosphate buffer solution (PBS) once, and ultra V protein block was applied and incubated for 10 min. Afterwards, the slides were rinsed with PBS twice, and the corresponding primary antibodies (Bcl-2, p53, and ER *β*) were applied. The slides were then incubated for 45 min, rinsed twice with PBS, and the primary antibody amplifier (secondary antibody) was applied. The slides were further incubated for 25 min, rinsed with PBS twice, and HRP polymer was added. The cycle of incubation for 25 min and rinsing twice with PBS was repeated. Sections were incubated for 5 min in diaminobenzidine (DAB) substrate, rinsed with PBS twice, counterstained with Haematoxylin, and rinsed with distilled water. Blueing solution was applied to the sections and rinsed, dehydrated, clear, and mounted for qualitative examination. For quantification, digital photographs obtained were imported unto the Image J software with specific plugins, and the results were normalized with the control group.

### 2.13. Statistical analysis

One-way analysis of variance (ANOVA) followed by Tukey's *post hoc* test was conducted using GraphPad PRISM 5 software (GraphPad Software, La Jolla, California, USA). Data were presented as mean ± SD with 5 replicates per group and *p* values < 0.05 were considered significant statistically.

## 3. Results

### 3.1. Change in body and testicular weight

Table 1 shows the body weight gain, paired testicular weight, and relative weight. Bisphenol F does not lead to a significant change in these parameters when compared with the age-matched control.

**Table 1:** Effect of BPF on body and testicular weight.

Parameters	Control	BPF-L	BPF-M	BPF-H	Control-R	BPF-LR	BPF-MR	BPF-HR
Body Weight Gain (g)	38.00 ± 0.2215	38.00 ± 0.2532	39.00 ± 0.2013	39.75 ± 0.2112	38.25 ± 0.2563	38.25 ± 0.2134	39.5 ± 0.2412	37.75 ± 0.2132
Paired Testicular Weight (g)	2.148 ± 0.2105	2.070 ± 0.2112	2.042 ± 0.3343	2.080 ± 0.2989	2.328 ± 0.3167	2.340 ± 0.1810	2.292 ± 0.3805	2.294 ± 0.3706
Relative Testicular Weight (g)	0.85 ± 0.1366	0.86 ± 0.185	0.90 ± 0.1517	0.91 ± 0.1142	0.84 ± 0.1460	0.82 ± 0.1289	0.88 ± 0.1797	0.90 ± 0.1775

Cntrl, control; BPF-L, bisphenol F low dose; BPF-M, bisphenol F medium dose; BPF-H, bisphenol F high dose; Cntrl-R, control recovery; BPF-L-R, bisphenol F low dose recovery; BPF-M-R, bisphenol F medium dose recovery; BPF-H-R, bisphenol F high dose recovery.

### 3.2. Histopathological findings

**Figure 1:**
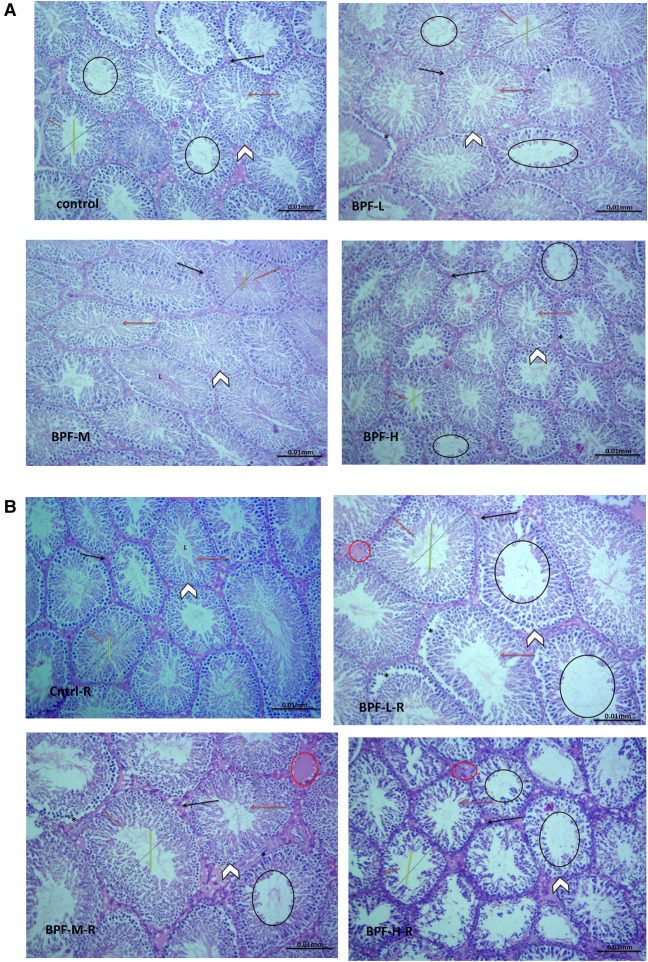
(**A**) Cntrl: the histoarchitecture appears normal. The seminiferous tubules appear normal in shape with germ cells at varying degree of maturation (arrow head). The Sertoli cells appear normal (red arrow). The lumen of the seminiferous tubules shows normal sperm cells (L). The interstitial space appears normal with normal Leydig cell mass (black arrow). BPF-L and BPF-M: The testicular histoarchitecture shows some distortions. The seminiferous tubules show germ cells at varying degree of maturation (arrow head) with some degenerated cell lines (*). The Sertoli cells appear normal (red arrow). The lumen of some seminiferous tubules shows scanty sperm cells (black circle). The interstitial space appears normal with normal Leydig cell mass (black arrow). BPF-H: The testicular histoarchitecture shows some distortions. The seminiferous tubules show germ cells at varying degree of maturation (arrow head) with some degenerated cell lines (*). The Sertoli cells appear scanty (red arrow). The lumen of most seminiferous tubules shows scanty sperm cells (black circle). The interstitial space appears normal with normal Leydig cell mass. Black span: diameter of the seminiferous tubules; red span: epithelial height; green span: diameter of the seminiferous lumen. Cntrl, control; BPF-L, bisphenol F low dose; BPF-M, bisphenol F medium dose; BPF-H, bisphenol F high dose; Cntrl-R, control recovery; BPF-L-R, bisphenol F low dose recovery; BPF-M-R, bisphenol F medium dose recovery; BPF-H-R, bisphenol F high dose recovery. (**B**) Cntrl-R: The histoarchitecture appears normal. The seminiferous tubules appear normal in shape with germ cells at varying degree of maturation (arrow head). The Sertoli cells appear normal (red arrow). The lumen of the seminiferous tubules shows normal sperm cells (L). The interstitial space appears normal with normal Leydig cell mass (black arrow). BPF-L-R and BPF-M-R: The testicular histoarchitecture shows some distortions. The seminiferous tubules show germ cells at varying degree of maturation (arrow head) with some degenerated cell lines (*). The Sertoli cells appear scanty (red arrow). The lumen of most seminiferous tubules appears widened with scanty sperm cells (black circle). The interstitial space appears widened with mild congestion (red circle). The Leydig cell mass appears normal (black arrow). BPF-H-R: The testicular histoarchitecture shows gross distortions. The seminiferous tubules show grossly altered spermatogenesis with coagulative necrosis in the germ cells (arrow head). The Sertoli cells are markedly scanty (red arrow). The lumen of almost all the seminiferous tubules appears widened with very scanty or no sperm cells (black circle). The interstitial space appears widened with mild congestion (red circle). The Leydig cell mass is reduced (black arrow). Black span: diameter of the seminiferous tubules; red span: epithelial height; green span: diameter of the seminiferous lumen. Cntrl, control; BPF-L, bisphenol F low dose; BPF-M, bisphenol F medium dose; BPF-H, bisphenol F high dose; Cntrl-R, control recovery; BPF-L-R, bisphenol F low dose recovery; BPF-M-R, bisphenol F medium dose recovery; BPF-H-R, bisphenol F high dose recovery.

The testicular histoarchitecture showed some abnormalities following treatment with BPF; the seminiferous tubules showed germ cells at varying degrees of maturation with some degenerated cell lines. The Sertoli cells appeared normal. Unlike the low and medium doses, the lumen of some seminiferous tubules in the animals treated with a high dose of BPF had scanty sperm cells compared with the age-matched control (Figure 1A). Surprisingly, cessation of BPF exposure did not reverse BPF-induced distortions in testicular histoarchitecture (Figure 1B).

Testicular histoarchitecture (using Cosentino's score) was significantly impaired across all the BPF-treated groups compared with their age-matched control, and this impairment was sustained in the recovery groups. Using the Johnson Biopsy score as an index for spermatogenesis, it was observed that BPF-H significantly impaired spermatogenesis compared with the control group, and this impairment was also observed in all the recovery groups. BPF led to a significant reduction in seminiferous tubular diameter and epithelial height and a significant increase in seminiferous luminal diameter compared with the age-matched control group. The decrease in seminiferous tubular diameter and epithelial height was observed at the medium and high doses of BPF treatment. Unlike epithelial height, where the observed decline was sustained in the recovery group, recovery restored the observed decrease in seminiferous tubular diameter. Furthermore, a significant increase in seminiferous luminal diameter was observed in the recovery groups in a dose-dependent manner (Table 2). In addition, a significant reduction was observed in both the Leydig cell and Sertoli counts in a dose-dependent manner and the observed decrease was sustained in the recovery groups.

**Table 2:** Effect of BPF on testicular histoarchitecture.

Parameters	Control	BPF-L	BPF-M	BPF-H	Control-R	BPF-LR	BPF-MR	BPF-HR
Testicular Biopsy Score	9.833 ± 0.4,082	9.000 ± 0.8,944	8.167 ± 0.9832	7.667 ± 0.8165^a^	9.833 ± 0.4082	6.500 ± 1.049^a,^*	6.667 ± 1.211^a,^*	3.167 ± 1.169^a,b,c,^*
Testicular Histoarchitecture	1.333 ± 0.5164	2.400 ± 0.8944^a^	2.500 ± 0.5477^a^	2.667 ± 0.5164^a^	1.333 ± 0.5164	2.667 ± 0.5164^a^	2.667 ± 0.5164^a^	3.667 ± 0.5164^a,b,c,^*
Epithelial Height (µm)	102.60 ± 7.102	87.61 ± 8.110	57.41 ± 1.443^a,b^	47.58 ± 7.326^a,b^	96.57 ± 7.898	63.27 ± 1.721^a^	54.54 ± 7.776^a,b^	49.22 ± 3.036^a,b^
Seminiferous Tubular Diameter (µm)	252.2 ± 29.89	260.1 ± 9.341	188.9 ± 24.12^a,b^	182.3 ± 24.98^a,b^	256.8 ± 21.12	277.6 ± 17.38	204.4 ± 11.81*	208.5 ± 4.359*
Seminiferous Luminal Diameter (µm)	72.07 ± 7.359	84.28 ± 7.16	82.00 ± 11.79	91.36 ± 13.61	71.46 ± 7.267	140.10 ± 9.490^a^*	170.1 ± 3.259^a,b,^*	188.3 ± 3.565^a,b,c,^*
Leydig count (cells/mm^2^)	4 ± 1	2.667 ± 0.5774	1.667 ± 0.5774^a^	1.333 ± 0.5774^a^	2.667 ± 0.5774	1.667 ± 0.5774^a^	1.667 ± 0.5774^a^	1.333 ± 0.5774^a^
Sertoli count/testis (×10^6^)	3.15 ± 0.0707	2.5 ± 0.1414^a^	2.15 ± 0.0707^a^	1.75 ± 0.0707^a,b,c^	3.2 ± 0.1414	2.165 ± 0.0,919^a^	1.89 ± 0.0141^a,b^	1.65 ± 0.707^a,b,c^

Cntrl, control; BPF-L, bisphenol F low dose; BPF-M, bisphenol F medium dose; BPF-H, bisphenol F high dose; Cntrl-R, control recovery; BPF-L-R, bisphenol F low dose recovery; BPF-M-R, bisphenol F medium dose recovery; BPF-H-R, bisphenol F high dose recovery. The testis was fixed in bouine solution, dehydrated with ethanol series, and cleared with toluene. The testes were embedded at room temperature and blocked in paraffin wax incubated overnight in a 600 C incubator. Afterward, hematoxylin and eosin (H&E) stain was applied to the testes’ 5 µm thick paraffin sections.

^a^ *p* < .05 vs. age-matched control.

^b^ *p* < .05 vs. low dose of BPF.

^c^ *p* < .05 vs. medium dose of BPF.

* *p* < .05 vs. groups with corresponding doses at *p* < .05 using one-way analysis of variance (ANOVA) followed by Tukey's *post hoc* test for pairwise comparison.

### 3.3. Male reproductive hormones

Table 3 shows the effect of BPF administration on male reproductive hormones. BPF caused a significant decrease in plasma GnRH, testosterone, FSH, and LH compared with animals in the control group. The reduction in plasma GnRH was only observed in the high-dose treated animals and all the BPF-treated recovery groups compared with the age-matched control counterpart. Plasma FSH and LH were significantly reduced following bisphenol F medium (BPF-M) treatment and bisphenol F high (BPF-H) treatment compared with the age-matched control group. These observed reductions were partially reversed by BPF cessation. In addition, BPF significantly reduced plasma testosterone levels in a dose-dependent manner compared with the age-matched control. Astonishingly, the observed decrease was worsened at recovery when compared with the age-matched control.

**Table 3:** Effect of BPF on reproductive hormones.

Parameters	Control	BPF-L	BPF-M	BPF-H	Control-R	BPF-LR	BPF-MR	BPF-HR
GnRH (mIU/ml))	12.07 ± 1.232	9.6 ± 1.751	8.27 ± 1.568	7.544 ± 1.291^a,b^	11.12 ± 1.002	7.207 ± 1.347^a^*	7.114 ± 1.101^a^*	7.278 ± 0.731^a^*
LH (mIU/ml)	5.648 ± 0.3496	5.331 ± 0.2109	2.901 ± 0.1376^a,b^	1.958 ± 0.1051^a,b,c^	5.716 ± 0.2385	5.131 ± 0.3231	2.817 ± 0.2172^a,b^	2.592 ± 0.1122^a,b,^*
FSH (mIU/ml)	4.796 ± 0.2147	4.756 ± 0.1886	4.374 ± 0.1876^a,b^	4.423 ± 0.2545^a,b^	4.782 ± 0.1943	4.138 ± 0.1056^a^*	3.902 ± 0.2675^a,b,^*	3.964 ± 0.2253^a,b,^*
Testosterone (ng/ml)	2.516 ± 0.1174	1.867 ± 0.1034^a^	1.32 ± 0.114^a,b^	0.8365 ± 0.0906^a,b,c^	2.479 ± 0.2228	1.771 ± 0.1252^a^	0.8595 ± 0.2144^a,b,^*	0.3908 ± 0.1283^a,b,c,^*

Cntrl, control; BPF-L, bisphenol F low dose; BPF-M, bisphenol F medium dose; BPF-H, bisphenol F high dose; Cntrl-R, control recovery; BPF-L-R, bisphenol F low dose recovery; BPF-M-R, bisphenol F medium dose recovery; BPF-H-R, bisphenol F high dose recovery.

^a^ *p* < .05 vs. age-matched control.

^b^ *p* < .05 vs. low dose of BPF.

^c^ *p* < .05 vs. medium dose of BPF.

* *p* < .05 vs. groups with corresponding doses at *p* < .05 using one-way analysis of variance (ANOVA) followed by Tukey's *post hoc* test for pairwise comparison.

### 3.4. Steroidogenic enzymes

**Figure 2:**
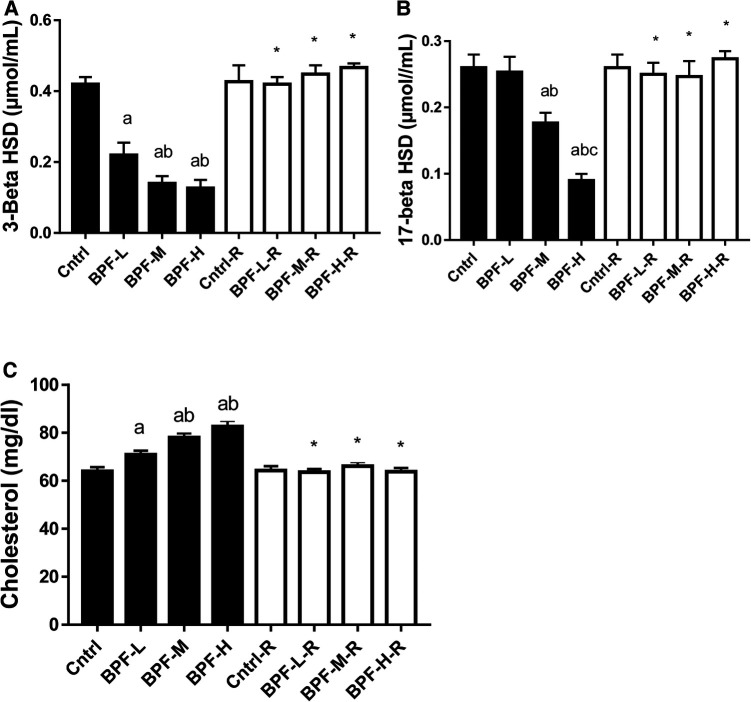
Effect of BPF on testicular (**A**) 3-beta HSD (**B**) 17-beta HSD (**C**) cholesterol. ^a^*p* < 0.05 vs. age-matched control, ^b^*p* < 0.05 vs. low dose of BPF, ^c^*p* < 0.05 vs. medium dose of BPF, *p < 0.05 vs. groups with corresponding doses at *p* < 0.05 using one-way analysis of variance (ANOVA) followed by Tukey's *post hoc* test for pairwise comparison. Cntrl, control; BPF-L, bisphenol F low dose; BPF-M, bisphenol F medium dose; BPF-H, bisphenol F high dose; Cntrl-R, control recovery; BPF-L-R, bisphenol F low dose recovery; BPF-M-R, bisphenol F medium dose recovery; BPF-H-R, bisphenol F high dose recovery.

There was a significant decrease in testicular 3-beta-hydroxysteroid dehydrogenase(3beta-HSD) and 17-beta hydroxysteroid dehydrogenase, as well as increased cholesterol in a dose-dependent manner when compared with the age-matched control (Figure 2). The observed decrease in these parameters were completely reversed following 28 days of withdrawal from BPF treatment.

### 3.5. Testicular injury markers

**Figure 3:**
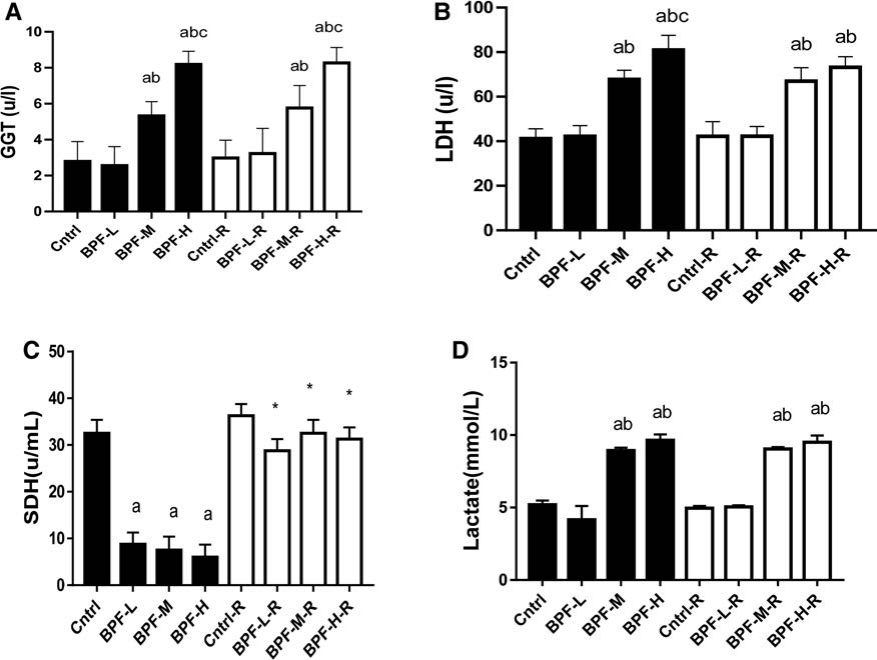
Effect of BPF on testicular (**A**) GGT (**B**) LDH (**C**) SDH (**D**) lactate ^a^*p* < 0.05 vs. age-matched control, ^b^*p* < 0.05 vs. low dose of BPF, ^c^*p* < 0.05 vs. medium dose of BPF, *p < 0.05 vs. groups with corresponding doses at *p* < 0.05 using one-way analysis of variance (ANOVA) followed by Tukey's *post hoc* test for pairwise comparison. Cntrl, control; BPF-L, bisphenol F low dose; BPF-M, bisphenol F medium dose; BPF-H, bisphenol F high dose; Cntrl-R, control recovery; BPF-L-R, bisphenol F low dose recovery; BPF-M-R, bisphenol F medium dose recovery; BPF-H-R, bisphenol F high dose recovery.

The activities of testicular GGT and LDH were significantly increased following the administration of medium and high doses of BPF. In contrast, the testicular SDH level was significantly decreased compared with the animals in the age-matched control group (Figure 3). Unlike the testicular SDH that was reversed following withdrawal, testicular GGT and LDH remained unchanged.

### 3.6. Inflammatory markers

**Figure 4:**
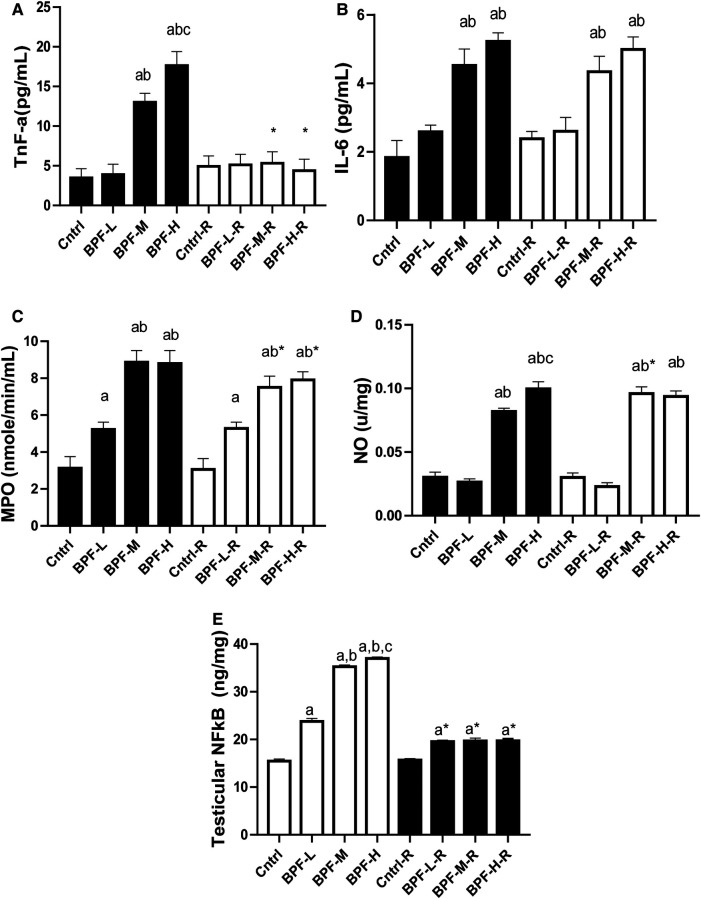
Effect of BPF on testicular (**A**) TnF-alpha (**B**) IL-6 (**C**) MPO (**D**) NO (**E**) nFkB. ^a^*p* < 0.05 vs. age-matched control, ^b^*p* < 0.05 vs. low dose of BPF, ^c^*p* < 0.05 vs. medium dose of BPF, *p < 0.05 vs. groups with corresponding doses at *p* < 0.05 using one-way analysis of variance (ANOVA) followed by Tukey's *post hoc* test for pairwise comparison. Cntrl, control; BPF-L, bisphenol F low dose; BPF-M, bisphenol F medium dose; BPF-H, bisphenol F high dose; Cntrl-R, control recovery; BPF-L-R, bisphenol F low dose recovery; BPF-M-R, bisphenol F medium dose recovery; BPF-H-R, bisphenol F high dose recovery.

Testicular TNF-alpha, IL-6, MPO, NO, and NFkB were significantly increased following BPF treatment, (Figure 4) compared with the age-matched control. The observed increase were not completely reversed following 28 days of withdrawal of BPF.

### 3.7. Oxidative stress markers

**Figure 5:**
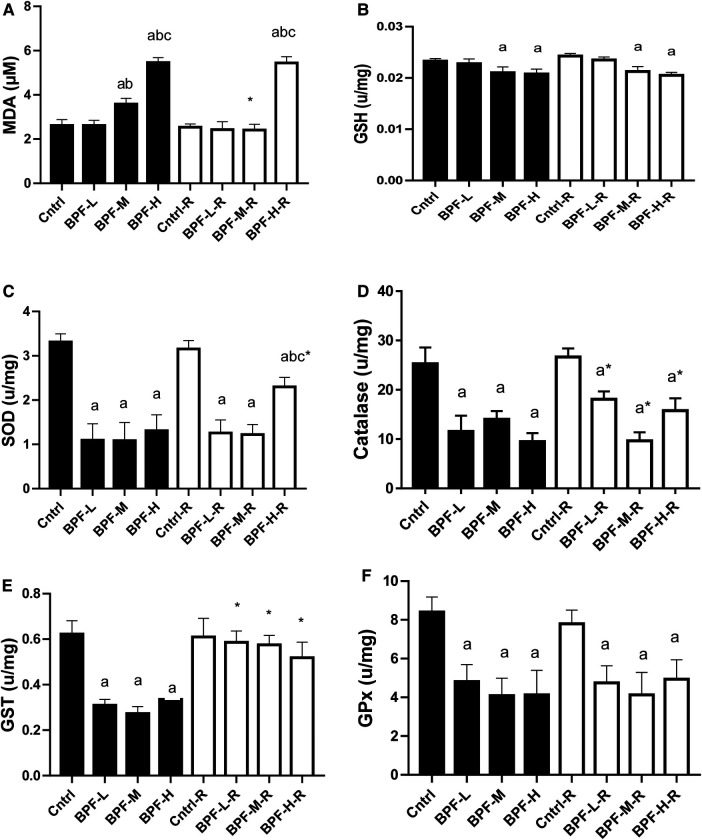
Effect of BPF on testicular (**A**) MDA (**B**) GSH (**C**) SOD (**D**) catalase (**E**) GST (**F**) GPx. ^a^*p* < 0.05 vs. age-matched control, ^b^*p* < 0.05 vs. low dose of BPF, ^c^*p* < 0.05 vs. medium dose of BPF, *p < 0.05 vs. groups with corresponding doses at *p* < 0.05 using one-way analysis of variance (ANOVA) followed by Tukey's *post hoc* test for pairwise comparison. Cntrl, control; BPF-L, bisphenol F low dose; BPF-M, bisphenol F medium dose; BPF-H, bisphenol F high dose; Cntrl-R, control recovery; BPF-L-R, bisphenol F low dose recovery; BPF-M-R, bisphenol F medium dose recovery; BPF-H-R, bisphenol F high dose recovery.

As shown in Figure 5, testicular MDA was significantly increased following medium and high doses of BPF treatment compared with the age-matched control counterpart. The observed increase in animals treated with medium-dose were reversed by withdrawal, and it remained unchanged in animals treated with high dose. Also, there was a significant reduction in testicular SOD, catalase, GSH, GST, GPX, and GST. While the significant decrease in testicular SOD was partially reversed in animals treated with a high dose of BPF following 28 days of recovery, the observed partial recovery in testicular catalase was at low and high doses. Unlike the other parameters, the recovery observed in testicular GST was complete, and it cut across all the recovery groups.

### 3.8. XO/UA signaling

**Figure 6:**
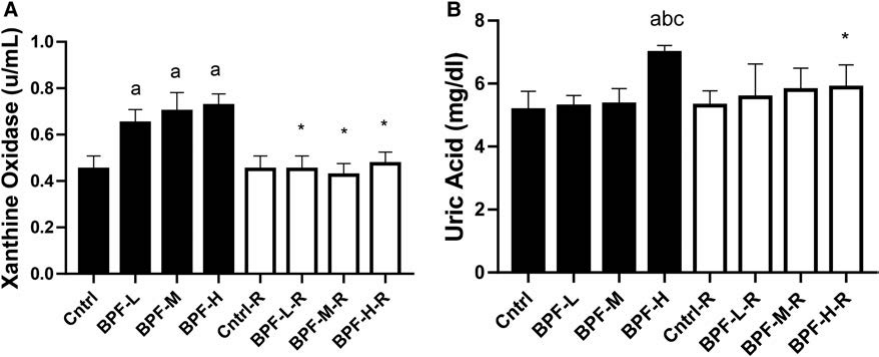
Effect of BPF on testicular (**A**) XO (**B**) UA. ^a^*p* < 0.05 vs. age-matched control, ^b^*p* < 0.05 vs. low dose of BPF, ^c^*p* < 0.05 vs. medium dose of BPF, *p < 0.05 vs. groups with corresponding doses at *p* < 0.05 using one-way analysis of variance (ANOVA) followed by Tukey's *post hoc* test for pairwise comparison. Cntrl, control; BPF-L, bisphenol F low dose; BPF-M, bisphenol F medium dose; BPF-H, bisphenol F high dose; Cntrl-R, control recovery; BPF-L-R, bisphenol F low dose recovery; BPF-M-R, bisphenol F medium dose recovery; BPF-H-R, bisphenol F high dose recovery.

Testicular xanthine oxidase and uric acid were significantly increased following BPF treatment (Figure 6). Unlike testicular xanthine oxidase, where the observed significant difference was noticed across all the groups, a significant increase in testicular uric acid was noticed in animals administered a high dose of BPF compared with the age-matched control. In addition, total recovery was observed in both testicular xanthine oxidase and uric acid following 28 days of recovery.

### 3.9. Apoptosis

**Figure 7:**
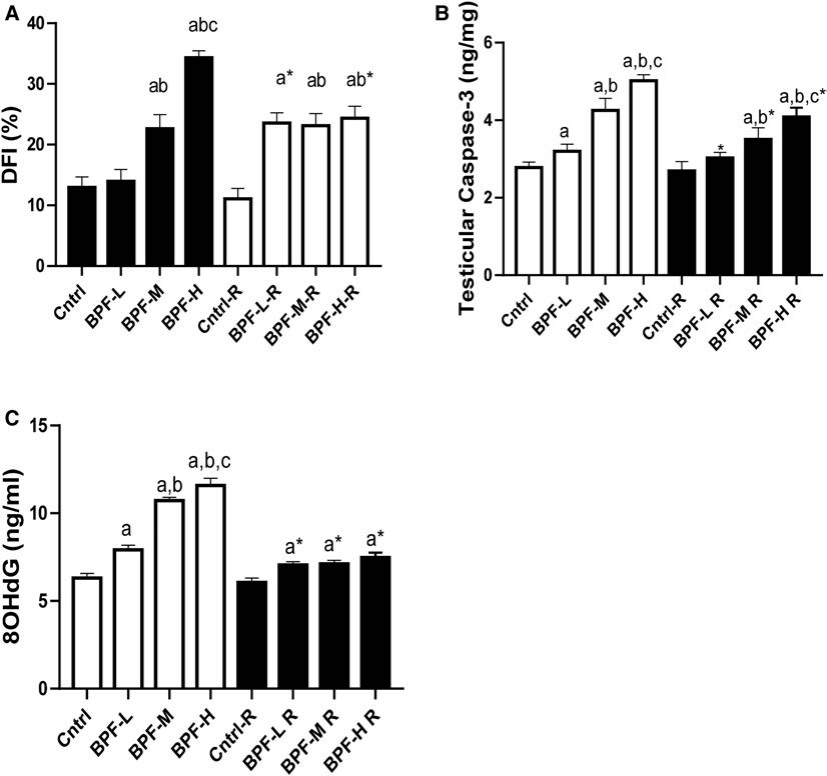
Effect of BPF on testicular DNA fragmentation Index (DFI). ^a^*p* < 0.05 vs. age-matched control, ^b^*p* < 0.05 vs. low dose of BPF, ^c^*p* < 0.05 vs. medium dose of BPF, *p < 0.05 vs. groups with corresponding doses at *p* < 0.05 using one-way analysis of variance (ANOVA) followed by Tukey's *post hoc* test for pairwise comparison. Cntrl, control; BPF-L, bisphenol F low dose; BPF-M, bisphenol F medium dose; BPF-H, bisphenol F high dose; Cntrl-R, control recovery; BPF-L-R, bisphenol F low dose recovery; BPF-M-R, bisphenol F medium dose recovery; BPF-H-R, bisphenol F high dose recovery.

**Figure 8:**
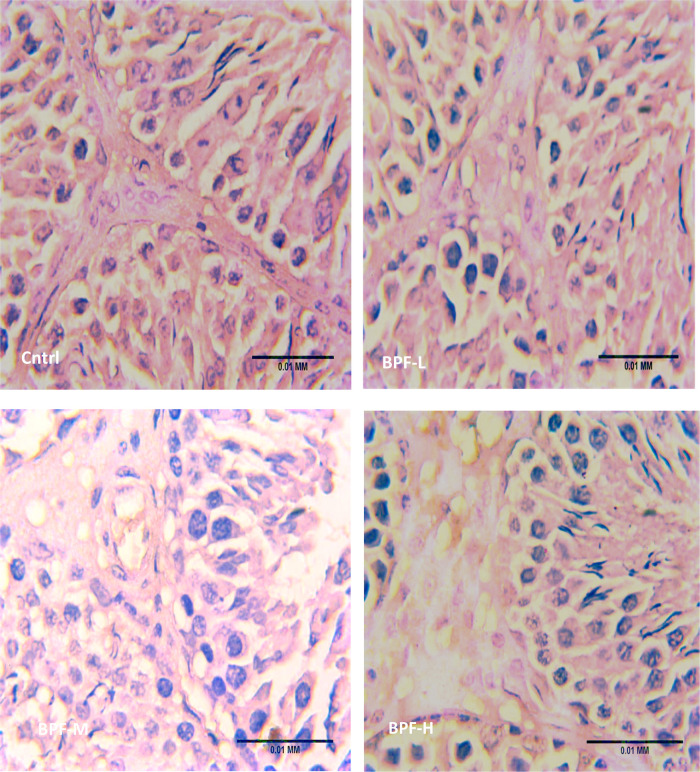
Effect of BPF on testicular BCl2. ^a^*p* < 0.05 vs. age-matched control, ^b^*p* < 0.05 vs. low dose of BPF, ^c^*p* < 0.05 vs. medium dose of BPF, *p < 0.05 vs. groups with corresponding doses at *p* < 0.05 using one-way analysis of variance (ANOVA) followed by Tukey's *post hoc* test for pairwise comparison.

**Figure 9:**
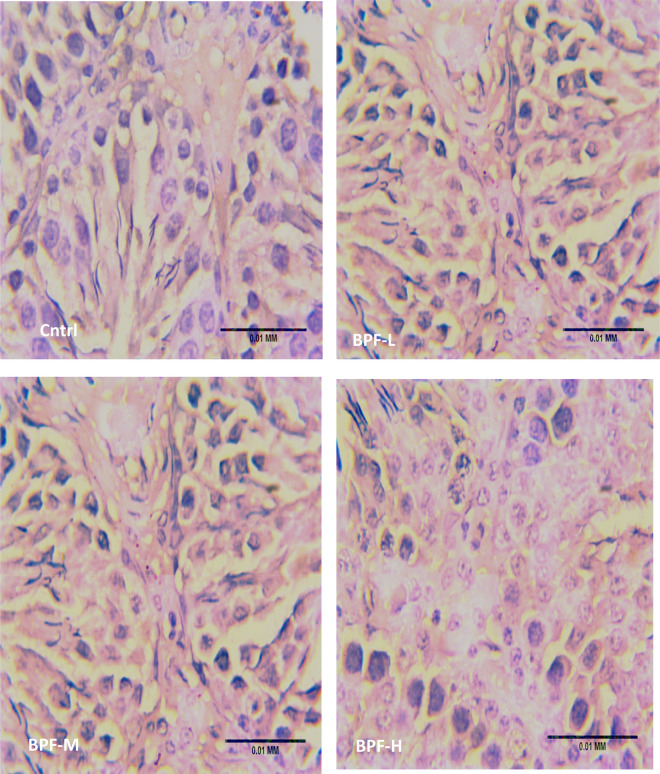
Effect of BPF on testicular p53. ^a^*p* < 0.05 vs. age-matched control, ^b^*p* < 0.05 vs. low dose of BPF, ^c^*p* < 0.05 vs. medium dose of BPF, *p < 0.05 vs. groups with corresponding doses at *p* < 0.05 using one-way analysis of variance (ANOVA) followed by Tukey's *post hoc* test for pairwise comparison. Cntrl, control; BPF-L, bisphenol F low dose; BPF-M, bisphenol F medium dose; BPF-H, bisphenol F high dose; Cntrl-R, control recovery; BPF-L-R, bisphenol F low dose recovery; BPF-M-R, bisphenol F medium dose recovery; BPF-H-R, bisphenol F high dose recovery.

Exposure to BPF led to a significant increase in testicular DFI, caspase 3, and 8HOdG (Figure 7). The increase in DFI was observed at medium and high doses, while the increase in caspase 3 and 8HOdG were dose-dependent. Furthermore, a significant decrease in testicular BCl2 (Figure 8) and an increase in p53 (Figure 9) were observed in a dose-dependent manner following BPF exposure. These observed differences were not completely reversed after the 28 days exposure free period.

**Figure:**
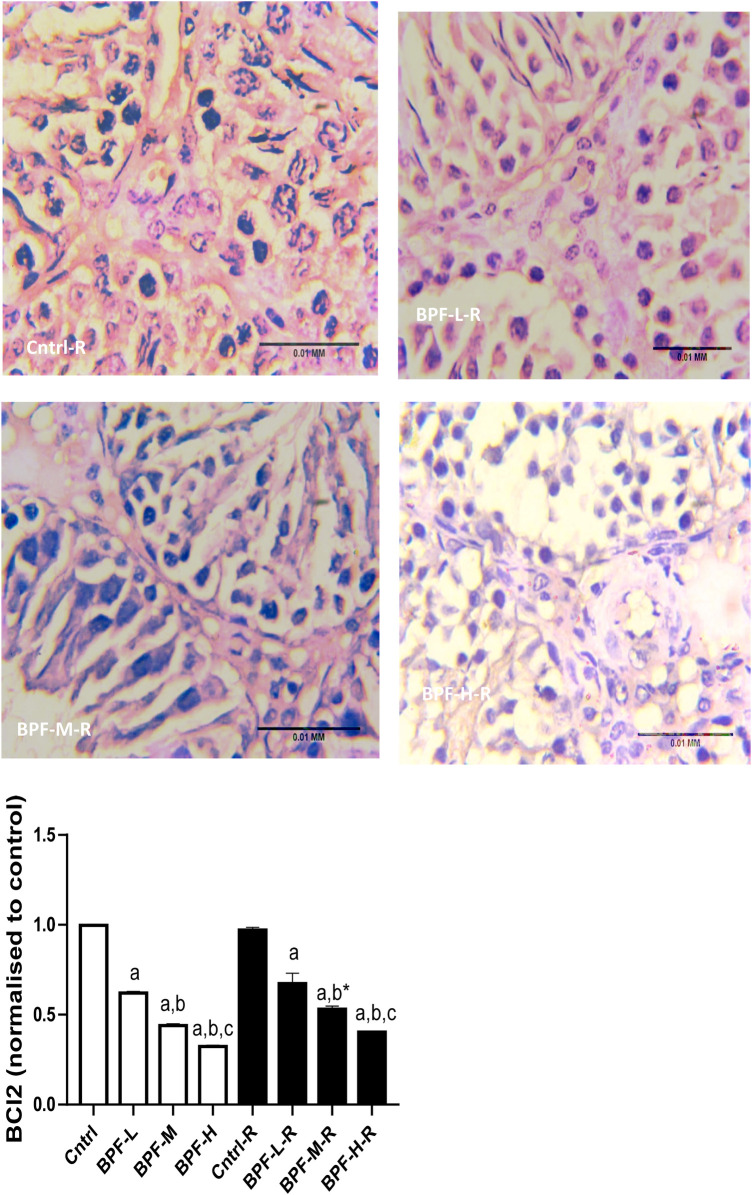


**Figure:**
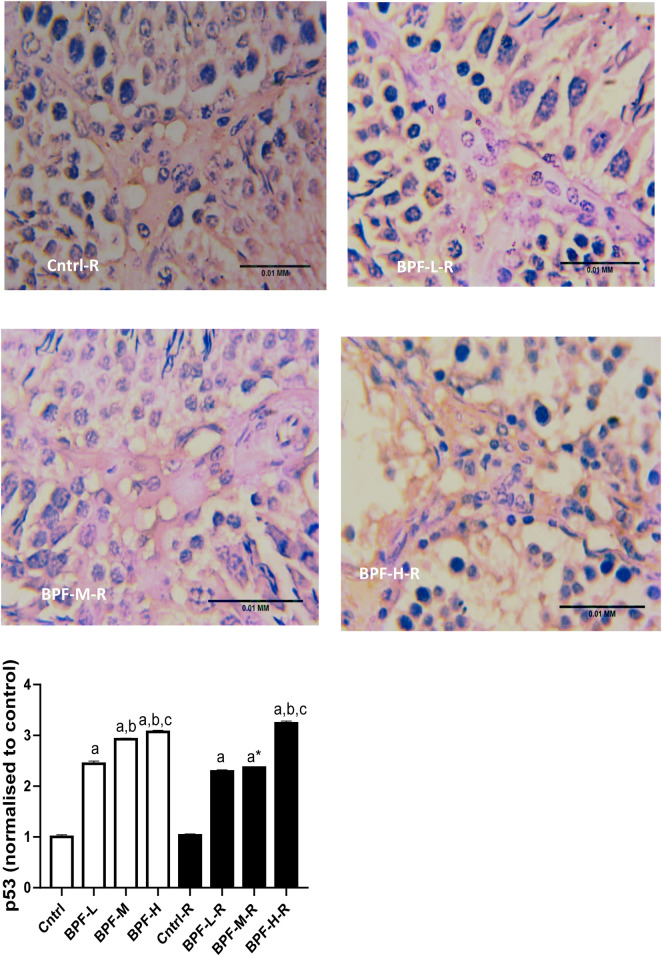


### 3.10. Oestrogen receptor

**Figure 10:**
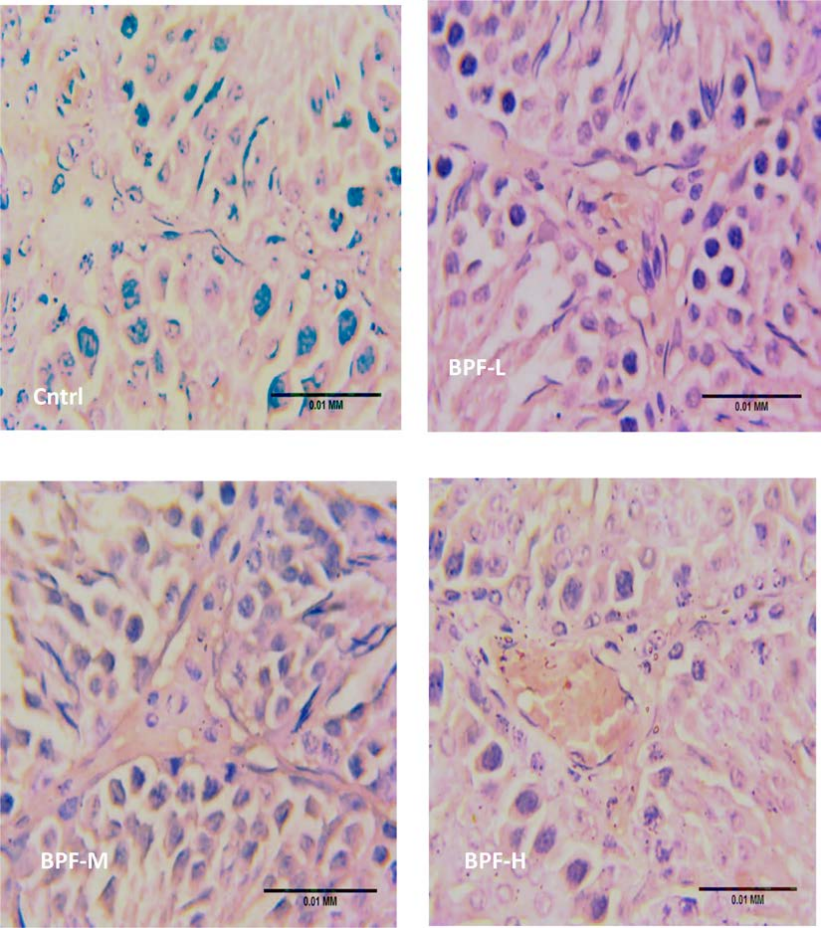
Effect of BPF on testicular ERβ. ^a^*p* < 0.05 vs. age-matched control, ^b^*p* < 0.05 vs. low dose of BPF, ^c^*p* < 0.05 vs. medium dose of BPF, *p < 0.05 vs. groups with corresponding doses at *p* < 0.05 using one-way analysis of variance (ANOVA) followed by Tukey's *post hoc* test for pairwise comparison. Cntrl, control; BPF-L, bisphenol F low dose; BPF-M, bisphenol F medium dose; BPF-H, bisphenol F high dose; Cntrl-R, control recovery; BPF-L-R, bisphenol F low dose recovery; BPF-M-R, bisphenol F medium dose recovery; BPF-H-R, bisphenol F high dose recovery.

Bisphenol F administration led to the overexpression of ERβ in a dose dependent manner and its withdrawal did not completely reverse the observed effect (Figure 10).

**Figure:**
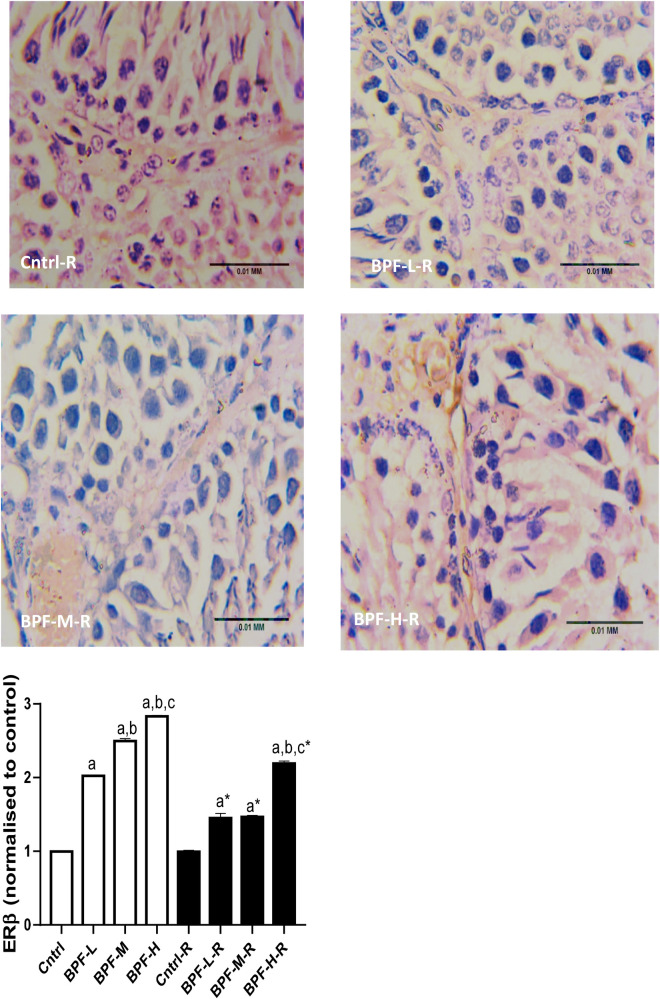


## 4. Discussion

Humans are constantly exposed to bisphenols; synthetic organic compounds used to manufacture hard plastic products for food packing, industrial material, personal hygiene products and dental sealants. The increasing rate of industrialisation and constant exposure to bisphenol has been implicated in the global rise in male infertility (30), which is majorly due to the release of endocrine disruptors into the environment. Bisphenol F is an endocrine disruptor, and little information is available about its effect on male reproduction. Although our previous findings have shown the impact of BPF on male reproductive hormones (4) and oxidative stress markers (15), there is no available information on the role of p53/BCl2 signaling and Erβ in BPF-induced testicular toxicity.

Bisphenol F treatment and its cessation did not significantly alter the body weight, paired testicular weight, or relative testicular weight compared with the age-matched control. This finding agrees with a previous study (31) that also observed no significant difference in body and testicular weight following BPF exposure.

BPF treatment distorted testicular histoarchitecture which was sustained after the recovery period. This agrees with previous findings that reported similar effect following the administration of BPF analogues (32, 33). The significant reduction in Johnsen's biopsy score (for spermatogenesis), epithelial height and seminiferous tubular diameter, as well as the increase in seminiferous luminal diameter, infer that BPF exerted a negative impact on the germ cell lines and damaged the mature sperm cells, thus reducing the germ cell layers and the volume of sperm cells in the lumen of the seminiferous tubules. This agrees with the study of (31), which reported a decrease in sperm counts following bisphenol F exposure. In addition, the observed reduction in Sertoli and Leydig cell counts also explains the BPF-induced reduction in matured luminal mature sperm cells and circulating testosterone, respectively. It is possible that BPF caused direct damage to the Sertoli and Leydig cells, thereby reducing their output. Since an adequate testosterone level is required for optimal Sertoli cell function and spermatogenesis, the reduced Sertoli cell count, epithelial height, and luminal sperm cell may be a consequence of BPF-induced suppression of testosterone.

BPF exposure significantly reduced the plasma GnRH (high dose), LH and FSH (medium and high dose), and testosterone (low, medium and high dose). BPF disrupted the physiological sequence of events along the hypothalamus-pituitary-testicular axis. Suppression of the hypothalamus-pituitary-testicular axis could significantly reduce the activity of GnRH-secreting cells and, hence, the hormone's plasma level. The consequent weak stimulatory effect of GnRH on the anterior pituitary causes impaired secretion of FSH and LH and thus a significant reduction in testosterone. These findings were similar to a previous study that reported a significant reduction in plasma testosterone, LH, and FSH following bisphenol F treatment (31).

BPF-induced suppression of steroidogenesis was associated with reduced steroidogenic enzymes, evidenced by the significant reduction in the activities of 3β-HSD at all doses of BPF and 17 β-HSD at medium and high doses, which was accompanied by increased cholesterol, the precursor of testosterone. These observations corroborate previous findings of (34), which reported similar observations following bisphenol A treatment. It is plausible to infer that BPF-induced impaired steroidogenesis was, at least in part, a consequence of BPF-induced downregulation of 3β-HSD and 17 β-HSD. Also, the reduced testicular cholesterol concentration may likely be due to impaired steroidogenic acute regulatory protein (StAR), responsible for cholesterol transportation to the inner mitochondrial membrane from the outer mitochondrial membrane. Bisphenol F exposure led to a significant drop in steroidogenic enzymes, which resulted in a decline in the circulating testosterone. The reduction in steroidogenic enzymes was restored in the animals in the recovery groups, which could result from the sustained decrease in circulating testosterone. Following cessation of BPF exposure, the persistent decline in circulating testosterone possibly triggered feedback on testicular steroidogenic enzymes (increase steroidogenic enzyme activities) in an attempt to maintain optimal testosterone production.

Furthermore, BPF treatment at a medium and high dose significantly increases testicular lactate, LDH, and GGT and causes a significant reduction in testicular sorbitol dehydrogenase (SDH). The significant increase in testicular lactate, an indicator of energy imbalance (35), could result from an observed increase in LDH. LDH is responsible for converting pyruvate to lactate during the anaerobic condition. It is important to note that there is no available data to compare our findings. Still, the significant increase in testicular LDH and lactate could be a compensatory adaptation to restore the observed BPF-induced testicular damage and oxidative stress. This agreed with a previous study by (36) that reported lactate to preserve germ cells by limiting the loss of spermatids and spermatocytes in rats. A primary index of the Sertoli role is GGT, and its activity is parallel to Sertoli cell replication and maturation (37). The significant increase in the activity of testicular GGT in animals treated with BPF in this present study suggests a less effective function of the Sertoli cell. SDH activities provide glycolysis and oxidative phosphorylation energy by converting sorbitol to fructose to form ATP (38). This study's observed unignorable reduction in SDH suggests that BPF treatment might lead to an energy imbalance in the sperm cell.

In this study, a significant increase in testicular MPO and NFkB was observed across all the doses of BPF. In contrast, a significant increase in testicular NO, TNF-α and IL-6 were observed after exposure to the medium and high dose of BPF. These findings agree with a previous study by Moon et al. (39), which reported a significant increase in pro-inflammatory markers after bisphenol-A administration. Notably, the significant increase in inflammatory markers could result from the increased generation of reactive oxygen species (40). The observed significant increase in testicular NO corroborates a previous study by Qiu et al. (41), that associated BPF treatment with a significant increase in NO. The significant increase in testicular NO may react with O2- to generate peroxynitrite, a potent oxidant that can decompose to produce reactive hydroxyl radical, eventually leading to testicular damage (42). The significant increase in testicular MPO might also contribute to the testicular toxicity observed in this study. This is in agreement with an investigation by Van der Veen et al. (43), that revealed that the MPO properties, which are autonomous of its enzymatic activity, and also oxidants that are MPO-derived, seem to take part in a series of events that assists the propagation and initiation of the inflammatory response and, as a result, are involved in tissue pathology in diseases depicted by oxidative stress and increase in inflammation.

The balance between the generation and scavenging reactive oxygen species (ROS) must be maintained under physiologic conditions. Under pathological conditions, excessive ROS are produced, altering the balance between ROS (pro-oxidants) production and removal (antioxidants). This condition is referred to as oxidative stress. In the testes of BPF-administered animals, oxidative stress was noticed. This was made evidenced by a significant increase in the level of testicular MDA at the medium and high doses of BPF and a significant reduction in testicular CAT, GST, SOD, and GPx at all doses of BPF exposure except testicular GSH, where the significant decrease was observed at the medium and high dose. The observed increase in pro-oxidants and decrease in antioxidants concurred with previous studies by Ullah et al. (31), & Olukole et al. (44), with similar findings following the administration of BPF and its analogue.

The increase in XO at all doses of BPF and Uric acid at high doses could significantly increase ROS levels since XO and lipid peroxidation have been linked together (16). However, it is important to note that UA may act as an antioxidant (45) and can curtail radical-forming systems (46). The pro-oxidant activity of UA can result from its reaction with other oxidants to produce radicals that majorly disrupt cellular activities (47). The lipophilic environment created due to the accumulation of lipids, in turn, creates an unfavourable environment for the antioxidant activity of UA (46), and the uric acid is thereby converted to oxidants by the oxidised lipids (49). The finding that BPF treatment significantly increased testicular uric acid levels suggests that its toxic effect on the testis could be due to the accumulation of uric acid. The significant increase in testicular UA may result in the activation of a pro-inflammatory response (50), resulting in a significant increase in testicular NO and MPO activity. Also, it may result in the observed oxidative stress following BPF treatment through increased generation of reactive oxygen species (48). Since BPF could stimulate both pro-inflammatory signaling and oxidative stress, it is logical to conclude that BPF mediates its gonadotoxic effect via XO/UA-dependent oxidative stress and inflammatory response.

The result from this study suggests that BPF-induced testicular apoptosis is through several interconnected pathways. The observed increase in the inflammatory response following BPF exposure and oxidative testicular damage could be the reason for the observed apoptosis (51). Furthermore, BPF-induced apoptosis can be traced to impaired p53/BCL-2 signaling. P53 has been referred to as the “guardian of the genome”, and it has been established to play its apoptotic role by inhibiting the activities of BCL-2, which has antiapoptotic functions (8, 10). Also, BPF increased testicular caspase 3, which is responsible for activating death protease that catalyses the cleavage of major cellular proteins (52).

These observations were associated with oxidative DNA damage and DNA fragmentation. This indicates that BPF stimulates the oxidation of guanines to generate 8OHdG, which labialised the glycosyl bond responsible for attaching guanines to adjacent ribose units, causing loss of guanine (53). This weakens the DNA backbone and causes the induction of localised strand breaks. The findings from this study that BPF induces apoptosis agreed with the reports from previous studies (54, 55).

Oestrogens are responsible for many physiological processes, such as growth and homeostasis in several tissues, by binding and activating the classical oestrogen receptors (ERα and ERβ) (56). Aside oestrogens, ERα and Erβ, can bind with different compounds, including BPF (57). Hence, BPF is referred to as “weak oestrogen” because it can bind with oestrogen receptors (with lesser affinity) and mimic oestrogens. Unlike BPA, other bisphenols have a higher affinity for ERβ (58). ERα is responsible for promoting cell proliferation, while the activation of ERβ has been associated with apoptosis. Oestrogens regulate spermatogenesis via ERα while spermatocyte apoptosis regulation is via ERβ (12). The overexpression of ERβ following BPF exposure is in tandem with the study of Matthews et al. (59) and suggests that BPF-induced apoptotic response could be via the binding of BPF with Erβ.

Surprisingly, BPF withdrawal did not completely reverse the observed disruptive effect of BPF on the testis. The testicular toxicity of BPF lingers beyond expectation, evidenced by unrestored testicular homeostatic balance within 28 days of BPF withdrawal. It is expected that withdrawal of BPF for 28 days would ameliorate BPF-induced gonadotoxicity; astonishingly, it did not. This may be due to its high affinity for fatty tissues. Bisphenols can accumulate in fatty tissues (60) such as the testis, thus exerting a prolonged effect even after cessation of exposure.

## 5. Conclusion

BPF instigates gonadotoxicity by promoting inflammatory response, oxidative stress, and apoptosis via the upregulation of the ERβ activities and distortion in p53/BCl2 signaling. These alterations were dose-dependent and minimal in BPF-L treatment. It is worth noting that cessation of BPF exposure did not completely restore the observed BPF-induced testicular toxicity.

## 6. Limitations

The histology of testes of animals treated with medium and high doses of BPF appear to have an increased number of vacuoles in the Leydig cells, and an Oil Red O stain would determine if these inclusions are lipid, and a PAS stain would test for carbohydrates. However, these special stainings were not done. This limitation in the present study opens a grey area for future exploration. Nonetheless, the in-depth testicular planimetry analysis and spermatogenic cell count provided in this study strengthen our findings on the disruptive activities of BPF on testicular histoarchitecture and testicular cells.

## Data Availability

The original contributions presented in the study are included in the article/Supplementary Material, further inquiries can be directed to the corresponding author/s.
